# Fostering Attachment Security: The Role of Interdependent Situations

**DOI:** 10.3390/ijerph17207648

**Published:** 2020-10-20

**Authors:** Francesca Righetti, Daniel Balliet, Catherine Molho, Simon Columbus, Ruddy Faure, Yaprak Bahar, Muhammad Iqmal, Anna Semenchenko, Ximena Arriaga

**Affiliations:** 1 Department of Experimental and Applied Psychology, VU Amsterdam, 1081BT Amsterdam, The Netherlands; d.p.balliet@vu.nl (D.B.); r.m.p.faure@vu.nl (R.F.); yaprakbahar5@gmail.com (Y.B.); muhammad.iqmal@hotmail.com (M.I.); anna.semenchenko@gmail.com (A.S.); 2 Institute for Advanced Study in Toulouse, 31080 Toulouse, France; catherine.molho@iast.fr; 3 Department of Psychology, University of Copenhagen, 1353 Copenhagen, Denmark; simoncolumbus@gmail.com; 4 Department of Psychological Sciences, Purdue University, West Lafayette, IN 47907-2081, USA; arriaga@purdue.edu

**Keywords:** attachment theory, situational interdependence, relationship satisfaction, trust, romantic relationships

## Abstract

This work adopts an Interdependence Theory framework to investigate how the features of interdependent situations that couples face in their daily life (i.e., situations in which partners influence each other’s outcomes) shape attachment security toward their current partners. An experience sampling study examined attachment tendencies and features of interdependent situations that people experience with their partner in daily life to predict satisfaction and trust in their relationship, and changes in attachment avoidance and anxiety toward their partner over time. Results revealed that encountering situations with corresponding outcomes (i.e., situations in which both partners have the same preferences) and with information certainty (i.e., situations in which there is clear knowledge of each partner’s preferences) assuage people’s insecurity. On the contrary, situations of mutual current and future interdependence (i.e., situations in which each person’s current or future outcomes are dependent on their partner’s behavior) undermined security for anxiously attached individuals. Power (i.e., the asymmetry in partners’ dependence) was not related to attachment security. This work underscores the importance of studying the role of the situations that partners experience in their daily life and the way they are related to relationship feelings and cognitions.

## 1. Introduction

While most people wish to feel secure and safe in their relationships, many individuals struggle with some form of attachment insecurity [1,2]. This manifests as frequent concerns about being loved, cared for and valued by others (attachment anxiety), and/or tendencies to avoid close emotional bonds and be skeptical about others’ trustworthiness (attachment avoidance). Such attachment insecurities are linked to several detrimental outcomes for personal and relational well-being [3,4,5,6]. Luckily, attachment insecurities are not set in stone; attachment tendencies can vary across partners [7] and change across the life course [8].

Recent theory and empirical research have examined how such changes occur and, importantly, how people can attain greater security in their current relationship [9]. Specifically, research has focused on what insecure individuals can do to foster a sense of attachment security [10] and how a relationship partner can help them in this process [9,11]. However, besides the role of the two interacting partners, as Social Psychology [12] and Interdependence Theory [13] emphasize, the features of the situations that people encounter with others can also play a major role in shaping people’s emotions, cognition, and behaviors. In this work, we adopt an Interdependence Theory framework to investigate how the features of the situations experienced in daily life with one’s romantic partner (i.e., correspondence of interests, information certainty, power, mutual dependence, and future interdependence; see Table 1 for definitions) are related to relationship outcomes for anxiously and avoidantly attached individuals, and how these situations can boost or undermine feelings of security toward one’s current partner.

### 1.1. Attachment Orientations

People who are securely attached feel personally valued and cared for by their partners. They trust their partners to be there for them in times of need, and are comfortable with closeness and intimacy [14]. In contrast, attachment insecurity in adults is expressed along two dimensions: anxious and avoidant attachment. Anxious individuals tend to have a history of inconsistent caregiver’s responses and they experience elevated fear of being rejected or abandoned. Their history of unreliable love and care induces them to be hypervigilant and overreact to anything that could be interpreted as a threat to their relationship. They often have elevated needs for reassurances of their partner’s love and commitment [15,16]. Avoidant individuals tend to have a history of neglect and unfulfilled interpersonal needs that induce them to withdraw from relationships, especially when relationships become emotionally intense. They tend to be overly self-reliant in an attempt to protect themselves from being hurt or disappointed by close others. Consequently, both anxious and avoidant individuals tend to distrust their partners [17] and to be less satisfied with their relationships [18] than secure individuals.

While there is some stability in attachment style across partners and situations, individuals may become more secure or insecure toward a specific partner depending on the experiences they encounter with them [7]. Recently, there have been significant theoretical [9] and empirical efforts [19,20] to understand how attachment insecurity can be modified and how people can gain more confidence in their current partner in the short and long term. For example, research has shown which strategies partners can enact to buffer a person’s immediate insecurity (e.g., deescalating negative emotions, being responsive to the individual’s need for connection or independence) [21,22] and which strategies may be effective in modifying insecurities in the long run (e.g., helping anxious individuals to attain their goals, showing gratitude to avoidantly attached individuals) [19,20,23]. However, until now, research has focused on partners’ behaviors and not much attention has been devoted to understand the role of the situations that partners encounter in their daily life. In fact, it has been proposed that certain situations may be likely to trigger insecurities while others may foster security [9]. Couples, for example, differ in the number of situations of correspondence vs. conflict of interests that they encounter in their daily life and these situations can affect people’s emotions, cognition, and ultimately their sense of security [24]. Which situations present opportunities and which present challenges for people’s security in their current relationship? Interdependence theory provides a key framework to investigate this research question by enabling in studying how the features of the interdependent situations that people experience in their dyadic life are related to people’s relationship outcomes.

### 1.2. Features of Interdependence

Interdependence Theory identifies six features to characterize key differences among interpersonal situations (i.e., correspondence of interests, information certainty, power, mutual dependence, future interdependence, and coordination; for definitions see Table 1) [25]. Five of the features have been shown to characterize how people subjectively perceive and understand everyday interactions (i.e., coordination is excluded) [26], and therefore became the focus of the current research. These are not specific interpersonal situations that people can readily grasp, such as being hurt by a partner or expressing gratitude. Rather, these “features” of situations are much more abstract, and they matter because they affect the benefits and costs that each person will take away from the interaction.

Situations are perceived along the dimension of *correspondence of interests*, that is the degree to which what is good for one person is also good for their partner. This could occur, for example, when both partners’ favorite activity is hiking in nature and they decide to spend their holiday hiking in the mountains together. This way, they both can do what they really enjoy the most. It could also happen when one partner prefers to cook and the other partner prefers to wash the dishes. By dividing the tasks in such a way, they both attain their preferred outcome. Situations also differ in *information certainty*, that is the degree to which partners know each other’s preferences and how their behaviors affect the other. For example, when couples clearly communicate their needs and feelings, people know what their partner wants and how to achieve it. On the contrary, when couples do not communicate well, when there is deception, or when couples encounter novel situations that are difficult to predict, people may have serious doubts about each other’s preferences and what the consequences of each behavior would be. Another important feature of the situation is *power*, or the extent that one person controls how good or bad an interaction will be for their partner. Couples confront this issue when one partner is at the mercy of a more able, resourceful, or desirable other individual. Consider competent individuals who care for a bedridden partner. Couples can detect such power differences and identify who has greater influence in an interaction, that is the degree to which one person controls and determines their own and their partner’s outcomes while the partner has no influence over them. Situations differ in *mutual dependence*, that is the degree to which both partners are dependent on each other to achieve what they want. In some situations, couples can be highly mutually dependent (such as when they decide to have children together and share responsibilities for them), in other situations they can be highly independent (such as when one person needs to make a work-related decision that does not affect their partner in any way). Finally, *future interdependence* characterizes the degree to which each person’s actions in the present moment can impact each other’s outcomes in the future. For example, buying a house together has extensive temporal consequences for both members of the couple, while deciding what to have for dinner tonight is unlikely to have long-term consequences for the couple. Obviously, each of these situational features vary in a continuous manner, such that many situations can contain a mixture of certain and uncertain information, or corresponding and conflicting interests.

### 1.3. Situations and Security

The features of interdependent situations that people encounter in their daily life may have an impact on people’s feeling of security with one’s partner because in those situations certain emotions and cognitions are more likely to be expressed. In fact, key features of the situations may activate people’s concerns about how partners may respond to those situations and, accordingly, may increase or decrease people’s fear about whether their partner will be responsive to their needs. When partners encounter a situation with corresponding interests, they are likely to feel in harmony, connected, and achieve good outcomes because both partners share the same desires [27]. While anyone can benefit from these types of situations, we posit that individuals who feel insecure in their relationship benefit especially from these situations. In fact, individuals who are insecurely attached to their partner have poorer expectations about what they can obtain from relationships [28,29]. When people’s outcomes exceed expectations, they are more likely to be satisfied than when people obtain just what they expect in their relationships [30,31,32]. Furthermore, achieving good outcomes in interactions can foster trust and the expectations that the partner will also provide good outcomes in the future [33]. Thus, we hypothesize that encountering correspondence of interests will be associated with positive relationship outcomes (i.e., increase relationship satisfaction and trust), especially in individuals high in attachment avoidance and anxiety toward their partner. Furthermore, the more people encounter situations of corresponding interests in their daily life, the more securely attached to their partner they will become over time.

Another important feature of interdependence that may play a key role in fostering security is information certainty. To the extent that people communicate well, are in familiar situations, and know what their partner wants, they should experience, on average, more pleasant interactions than when there is doubt, uncertainty, and room for misunderstandings. Given that insecurely attached individuals tend to have negatively biased perceptions of others and of their emotions [29,34], they may be especially likely to benefit from situations with clear information that prevent them from adopting a negative perspective. Thus, we hypothesize that encountering situations with high information certainty will be associated with positive relationship outcomes in people high in attachment avoidance and anxiety toward their partner. Furthermore, the more people encounter situations with information certainty in their daily life, the more securely attached to their partner they will become over time.

Having power in the situation, and being able to control one’s own and the other person’s outcomes, may also assuage people’s insecurity. This may be especially true for anxiously attached individuals who chronically tend to fear abandonment and rejection [35]. The awareness that their partner needs them more than they need their partner should make them feel safe and in control. Thus, we hypothesize that encountering situations with high power will be associated with positive relationship outcomes for individuals high in attachment anxiety toward their partner. Furthermore, the more that anxious people encounter situations with power in their daily life, the more securely attached to their partner they will become over time.

Finally, we do not make any a priori predictions regarding mutual dependence and future interdependence. In fact, on the one hand, having high current (and future) dependence could possibly activate people’s anxiety because they need to rely on their partner and they doubt that their partner will provide good outcomes. However, on the other hand, it may also be possible that individuals high in attachment anxiety may benefit from being in situations in which their partner is also dependent on them in the short and long-run. In fact, in these situations anxious individuals may feel “needed”, which should reassure them that the partner is not going to reject or leave them.

To test these ideas, we used an experience sampling study with couples. At Intake participants reported their attachment orientation toward their current partner. In the experience sampling, every two hours participants were asked to report the features of the last situation that they encountered with their partner. In this way, we examined how the features of the interdependence interacted with people’s attachment orientation to predict current relationship outcomes (i.e., relationship satisfaction and trust). Attachment orientation toward their partner was also re-assessed about ten days after the completion of the experience sampling. We could then also examine whether the average levels of correspondence of interests, information certainty, power, mutual dependence, and future interdependence that they had encountered during the experience sampling study related to changes in partner-specific attachment orientation shortly afterwards.

## 2. Materials and Methods 

### 2.1. Participants 

We used a panel agency to collect data from a Dutch community sample of romantic couples (*N* = 278 individuals; 50.7% male; *M_age_* = 32.04 years, SD*_age_* = 13.56, range 18–79). The sample size was based on typical sample sizes in relationship studies taking into account financial and practical constraints. Participants were recruited via online panels and were paid differently for the different parts of the study (on average, they earned €63.65). All participants lived in the Netherlands. The reported median monthly net income was €1200–1399, and a significant proportion reported monthly net incomes above €2500 (14.0%); 29.5% of the participants had a university education, 32.7% had completed vocational training, and 35.6% had completed secondary education. All couples were heterosexual, except for one homosexual couple. Couple’s relationship length was on average 7.70 years (SD = 10.50).

### 2.2. Procedure

Couples came to the laboratory for an intake session, and after signing an informed consent they were separated and asked to fill in some questionnaires, among which was one concerning their attachment orientation. At the end of the intake session they were given instructions about the experience sampling procedure. In the experience sampling, for seven consecutive days, participants received seven messages a day between 08:00 and 22:00. This window was divided into seven blocks of 2 h, and participants received a message at a random time within each block (with a minimum of 45 min between messages). Survey links remained open for 45 min. Participants were asked whether they had experienced a situation with their partner since responding to the last questionnaire. If so, they were asked to report on the last situation they had experienced with their partner. If not, they were redirected to other questions not relevant for the present investigation. The overall response rates were 81.6%, and median per-subject response rates were even higher (89.8%). We obtained 6766 responses regarding situations with one’s partner. About ten days after the end of the experience sampling phase, participants completed a follow-up survey which also contained an assessment of their attachment orientation. The overall response rate was 75.18% (203 complete responses). Ethical approval for the study was obtained from the Scientific and Ethical Review Board of the VU Amsterdam (VCWE-2017-003). For further details about the procedure see [27].

### 2.3. Material

#### 2.3.1. Intake Survey

During the laboratory intake, participants completed several questionnaires and performed several tasks. Relevant for the current investigation, participants completed a measure of anxious and avoidant attachment orientations which was partner specific, that is, on how they felt in relation to their current partner (Experience in Close Relationships (ECR)-Relationship Structures; 5 items for avoidant; e.g., “I don’t feel comfortable opening up to my partner”; α = 0.66; 3 items for anxious; e.g., “I often worry that my partner does not really care for me”; α = 0.85) on a 7-point scale (1 = completely disagree, 7 = completely agree) [7]. Higher scores on each indicated higher avoidance and anxious attachment, respectively.

#### 2.3.2. Experience Sampling Reports

First, participants were asked to think about and describe the last situation that they experienced with their partner (since the previous signal). For each reported situation, participants were required to complete the 10-item Situational Interdependence Scale [26]. The 10-item version of the SIS has been designed to measure situation-specific perceptions of the five interdependence features and has been shown to be reliable and valid [26,27]. All items, excluding power, were answered on five-point Likert-type scales (1 = completely disagree, 5 = completely agree). For each situation, participants reported their correspondence of interests (two items; “We can both obtain our preferred outcomes” and “Our preferred outcomes in this situation are conflicting”, second item is reverse coded; α = 0.69), their information certainty (two items; “We both know what the other wants” and “I don’t think the other knows what I want”; second item is reverse coded; α = 0.70), their mutual dependence (two items; “What each of us does in this situation affects the other” and “Whatever each of us does in this situation, our actions will not affect the other’s outcomes”, second item is reverse coded; α = 0.62), future interdependence (two items; “How we behave now will have consequences for future outcomes” and “Our interaction has no effect on future behavior in interactions with each other”, second item is reverse coded; α = 0.71), and power (two items; “Who has the most impact on what happens in this situation?” and “Who has the least amount of influence on the outcomes of this situation?” second item is reverse coded; α = 0.84, 1 = totally the other, 5 = totally myself). Afterwards, participants were asked to report on how they felt about their relationship at the present moment. Specifically, they reported their current relationship satisfaction (“I am satisfied with my relationship”) and current trust in their partner (“I trust my partner”) on a five-point Likert-type scales (1 = completely disagree, 5 = completely agree).

#### 2.3.3. Follow-Up Survey

About ten days after completing the experience sampling phase of the study, participants completed the same partner-specific attachment orientation scale that was administered on the Intake survey (avoidant α = 0.85, anxious α = 0.85).

## 3. Results

### 3.1. Analysis Strategy

The statistical analyses were performed using multilevel analyses given that the data included observations that were not independent and were clustered within the same person and couple (i.e., each participant provided multiple assessments across time, and pairs of participants were nested within couples). We used a two-level cross-model in which participants and daily measurements within participants were treated as crossed and nested within the dyad [36]. Intercept terms were treated as random, and slopes as fixed effects. Dyads were treated as indistinguishable [36].

Each interdependence feature was examined one at a time. Regression models predicted daily levels of relationship satisfaction and trust from daily reports of an interdependence feature, intake levels of attachment anxiety and attachment avoidance, and interaction terms between an interdependence feature and attachment orientations. Significant interactions were interpreted by examining the simple slope of each feature for individuals high (+1 SD) vs. low (−1 SD) in avoidantly attached or anxiously attached individuals. As is typically done for experience sampling data, each person’s daily report of an interdependence feature was scaled for that person’s average or “typical” level (i.e., interdependence feature variables were person-centered to examine within-person variations). This enabled us to investigate several questions pertaining to interdependence features, such as, for example, whether a person feels more satisfied or experiences higher trust in situations of greater correspondence with their partner, relative to what the person typically feels, and whether this differs among insecure versus secure individuals.

Furthermore, additional analyses examined changes in attachment orientations ten days after the experience sampling. As is typically done for analysis of change across time, time-lagged multilevel analyses tested whether a person’s mean report of each interdependence feature experienced during the experience sampling phase predicted that person’s level of attachment anxiety and avoidance at the follow-up, while controlling for the initial level of anxiety and avoidance (i.e., predicting a person’s attachment security after the experience sampling phase while controlling for that person’s attachment security before the experience sampling phase). This enabled us to investigate additional issues pertaining to interdependence features, such as whether individuals who generally experience high levels of corresponding interests and information certainty in their daily interactions with their partner become more securely attached over time.

Supplementary Materials (Tables S1–S4) includes the correlations between features of interdependence and the results of the full model with all the interdependence features, attachment orientations, and their interactions as predictors (i.e., 17 simultaneous predictors).

### 3.2. Key Findings

#### 3.2.1. Experience Sampling Reports 

Results are presented in Table 2 and Table 3. As expected, for both relationship satisfaction and trust, there was a significant interaction between avoidant attachment and corresponding interests. Simple slope analyses revealed that individuals high in attachment avoidance (+1 SD) experienced higher relationship satisfaction and trust when encountering situations with more corresponding interests than they typically do (*b* = 0.10, SE = 0.02, 95% CI [0.06, 0.13], *p* < 0.001, and *b* = 0.07, SE = 0.01, 95% CI [0.04, 0.10], *p* < 0.001, respectively). These effects were weaker for individuals low in attachment avoidance (−1 SD; *b* = 0.06, SE = 0.01, 95% CI [0.04, 0.09], *p* < 0.001, and *b* = 0.04, SE = 0.01, 95% CI [0.01, 0.06], *p* = 0.001, respectively). The interactions with anxious attachment were not significant.

As expected, for relationship satisfaction, there was a significant interaction between avoidant attachment and information certainty. Specifically, individuals high in attachment avoidance experienced higher relationship satisfaction when encountering situations with more information certainty (+1 SD; *b* = 0.09, SE = 0.02, 95% CI [0.06, 0.13], *p* < 0.001) than individuals low in attachment avoidance (−1 SD; *b* = 0.05, SE = 0.01, 95% CI [0.02, 0.08], *p* < 0.001). For trust, this interaction was not significant. There were significant interactions between anxious attachment and information certainty for both relationship satisfaction and trust. Specifically, individuals high in attachment anxiety (+1 *SD*) experienced higher relationship satisfaction and trust when encountering situations with more information certainty (*b* = 0.09, SE = 0.02, 95% CI [0.04, 0.13], *p* < 0.001, and *b* = 0.06, SE = 0.02, 95% CI [0.04, 0.10], *p* = 0.003, respectively) than individuals low in attachment anxiety (−1 SD; *b* = 0.04, SE = 0.02, 95% CI [0.00, 0.08], *p* = 0.042, and *b* = 0.03, SE = 0.02, 95% CI [0.01, 0.06], *p* = 0.054, respectively).

For both relationship satisfaction and trust, there was also a significant interaction between mutual dependence and anxious attachment. Specifically, individuals high in attachment anxiety (+1 *SD*) experienced lower relationship satisfaction and trust when encountering situations with more mutual dependence (*b* = −0.05, SE = 0.02, 95% CI [−0.09, −0.01], *p* < 0.001, and *b* = −0.02, SE = 0.02, 95% CI [−0.06, 0.01], *p* = 0.225, respectively), than individuals low in attachment anxiety (−1 SD; *b* = −0.01, SE = 0.02, 95% CI [−0.04, 0.02], *p* = 0.517, and *b* = 0.01, SE = 0.01, 95% CI [−0.02, 0.03], *p* = 0.735, respectively). The interactions with avoidant attachment were not significant. Furthermore, for relationship satisfaction, there was a marginally significant interaction between future interdependence and anxious attachment. Specifically, individuals high in attachment anxiety (+1 *SD*) experienced lower relationship satisfaction when encountering situations with future interdependence (*b* = −0.05, SE = 0.02, 95% CI [−0.09, −0.01], *p* = 0.013), than individuals low in attachment anxiety (−1 SD; *b* = −0.03, SE = 0.02, 95% CI [−0.06, 0.01], *p* = 0.113). For trust, none of the interactions were significant. Contrary to the hypotheses, power did not interact with attachment orientations for either outcome.

#### 3.2.2. Follow-Ups

In the follow-up, we examined whether changes in attachment orientations occurred over time as a function of the daily situational features that individuals experienced with their partner. Results revealed that individuals who experience greater correspondence of interests with their partner during the experience sampling phase became less avoidantly attached and less anxiously attached ten days later, controlling for Intake-levels of attachment avoidance and anxiety (*b* = −0.33, SE = 0.12, 95% CI [−0.57, −0.08], *p* = 0.009, and *b* = −0.41, SE = 0.16, 95% CI [−0.72, −0.09], *p* = 0.013, respectively). Moreover, individuals who experienced greater information certainty became less avoidantly attached and less anxiously attached ten days later, controlling for Intake levels of attachment avoidance and anxiety (*b* = −0.30, SE = 0.12, 95% CI [−0.54, −0.06], *p* = 0.015, and *b* = −0.50, SE = 0.16, 95% CI [−0.81, −0.19], *p* = 0.002, respectively). Mutual dependence, future interdependence, and power did not predict changes in attachment orientations (all *p*s > 0.10).

## 4. Discussion

The present work investigates the role played by the situations that couples experience in their daily life in shaping attachment security toward their current partner. As hypothesized, avoidantly attached individuals especially benefitted from experiencing situations of corresponding interests and situations high in information certainty with their partner. In fact, encountering situations of corresponding interests and situations high in information certainty was associated with greater relationship satisfaction and trust for everyone, and this relationship was especially strong for avoidant individuals. Furthermore, encountering situations of corresponding interests and high in information certainty in their daily life predicted reduced attachment avoidance toward their partner over time.

Anxiously attached individuals especially benefitted from encountering situations high in information certainty. While information certainty was positively associated with relationship satisfaction and trust overall, this association was especially strong for individuals high in attachment anxiety. Furthermore, encountering situations high in information certainty in the experience sampling predicted reduced attachment anxiety toward their partner over time. Similarly, encountering situations of corresponding interests in the experience sampling predicted lower attachment anxiety two weeks later. Interestingly, anxiously attached individuals experienced worse outcomes when encountering situations of current and future mutual dependence. Whereas secure individuals were not affected by current mutual dependence and future interdependence, anxiously attached individuals experienced lower relationship satisfaction when encountering these situations. These findings were not predicted a priori, but they may reflect the idea that, for anxious individuals, moments in which they may be strongly reliant on a partner, now or in the future, will activate worries and fears that their partner may not be responsive to their needs. They are also consistent with the notion that anxiously attached individuals may become more secure in their relationship when they can strengthen their independence and self-reliance [9]. We need to acknowledge, though, that these negative outcomes may be driven by the anxious individuals’ own dependence on their partner, rather than mutual dependence; our measure was not well equipped to differentiate between the two. Contrary to expectations, power was not related to feelings of security. One possibility for this null finding may be that power also enhances people’s feeling of responsibility toward their partner [37], activating insecurities and doubts about the best course of action for the relationship.

This work underscores the importance of the situations that couples encounter in their daily life in predicting people’s feelings about their relationship. So far, relationship science has given much emphasis to partners’ behaviors, but partners’ actions are strongly tied to the situations that they experience. For example, while it may be very easy to behave in a trustworthy manner in situations of corresponding interests, it may be way more challenging to do so in situations of conflict of interests [27,33]. Interdependence theory [13,25] provides the ideal framework to investigate how the properties of social situations that partners experience shape relationship dynamics and dispositions. The present work also contributes to the literature on interdependence by revealing how certain individuals may react differently to the social situations that they experience. For example, the current findings show that anxiously and avoidantly attached individuals may be more sensitive to certain features of the situation than securely attached individuals.

Our findings have important theoretical and practical implications for the study of attachment security and how it can be fostered in romantic relationships. Results revealed that situations with high information certainty induce both anxiously and avoidantly attached individuals to feel more satisfied and trusting. Future research should focus on the factors that may promote such information certainty. For example, good communication of preferences and needs from both partners may be key to instilling a sense of security [38]. Being familiar with situations and knowing one’s partner and what to expect from them may also prevent insecure individuals to adopt negatively biased perceptions that may perpetuate their insecurities [34]. Previous research has shown that attachment insecurity decreases with age [8], which might partly be explained by the fact that partners come to know each other well over time and become experienced with a broad range of situations together, leaving less room for interpersonal noise [39] and negative interpretations [40].

Another feature of interpersonal situations that seems to play an important role in reducing attachment insecurity is having corresponding interests. Encountering situations of conflict of interests with one’s partner can be highly distressing for the individual and the relationship [41] and may be likely to result in conflicts [42]. In these situations, people are likely to feel vulnerable because good outcomes for both partners are difficult to achieve [33]. On the contrary, when partners encounter situations of corresponding interests they can enjoy their interdependence while achieving mutually good outcomes. These situations seem to be especially beneficial for avoidantly attached individuals, but when repeated over time they also seem to reduce insecurity for anxiously oriented individuals [9]. Future research should investigate which couples are more likely to encounter these situations in their life (e.g., couples with similar interests and values) and what partners can do to selectively try to experience situations in which their interests align rather conflict.

Some limitations of the current work need to be acknowledged. First, data obtained from daily reports in the experience sampling phase were cross-sectional, limiting our ability to make firm causal conclusions. However, our lagged analyses of the follow-up also showed that the average levels of corresponding interests and information certainty in the experience sampling were associated with later changes in attachment orientations toward current partners above and beyond the initial levels of attachment anxiety and avoidance. Second, we assessed each person’s perception of their interdependent situation with their partner, rather than the objective situation that the couple confront. However, previous research has shown that such perceptions tend to track objective features of reality, so there typically is a correspondence between the objective situation and the perceived one [26,27]. Yet it is the subjective construal of the situation, rather than its objective features, that should exert a strong influence on people’s emotions, cognitions, and behavior [43,44,45,46]. Furthermore, although insecure individuals may have biased perceptions of interpersonal situations, perhaps perceiving them more negatively than secure individuals do [29], the within-person analyses took into account each person’s own general way of perceiving things and thus adjusted for negativity or other biases. This analysis isolated the role of perceiving more (or less) corresponding interests as compared to each person’s typical (mean) perceptions in predicting trust and satisfaction.

Some strengths should also be acknowledged. The present work is one of the first attempts to study the impact that the features of the situations that couples encounter in their daily lives have on relationship dispositions and outcomes. Furthermore, the research questions were tested in an ecologically valid manner by sampling random situations from a large sample of couples’ everyday lives and asking participants to report on them and on their current feelings. This method reduces memory bias and allows for reports of situations close to their occurrence, while maximizing statistical power and precision [47]. Additionally, besides assessing current relationship outcomes shortly after the situation in the experience sampling, we were also able to conduct time-lagged analyses to examine actual changes in attachment orientation toward their current partner above and beyond initial level of attachment insecurity. Finally, although the sample was skewed toward younger individuals, it was heterogeneous in terms of age, education, and income.

## 5. Conclusions

The study of interdependent situations can provide invaluable insights into the factors that promote attachment security in a romantic involvement. Our investigation revealed that experiencing situations of corresponding interests and information certainty foster relationship satisfaction and trust, and that individuals who are insecurely attached to their partner are most likely to benefit from such features of interdependence. Furthermore, repeatedly encountering situations of corresponding interests and information certainty promoted positive changes in attachment security toward their partner over time. On the contrary, situations of mutual dependence seemed to exacerbate people’s anxiety. Although sometimes people have little control over the situations that they encounter in their lives, other times people can selectively choose to enter, modify, or avoid certain situations. Our findings reveal which situations people should strive for if they want to improve feelings of safety and connection in their intimate relationships.

## Figures and Tables

**Table 1:** Definitions of the features of interdependent situations.

Dimension	Definition
Correspondence of interests	The degree to which the best outcome for one individual is also the best outcome for their partner (vs. conflict of interests).
Information certainty	The degree to which a person knows their partner’s preferences and how each person’s actions influence each other’s outcomes.
Power	The degree to which an individual determines their own and their partner’s outcomes, while the partner does not have much influence.
Mutual dependence	The degree to which both individuals mutually influence each other’s outcomes.
Future interdependence	The degree to which both individuals’ actions influence both individuals’ future outcomes.

**Table 2:** Predicting reports of relationship satisfaction from levels of each interdependence feature, intake levels of attachment orientations, and their interactions.

	*b*	SE	95% CI	*p*
Correspondence	0.04	0.02	0.01, 0.08	0.025
Avoidant	−0.23	0.04	−0.30, −0.15	0.001
Anxious	−0.07	0.02	−0.11, −0.02	0.004
Correspondence X Avoidant	0.02	0.01	0.01, 0.04	0.026
Correspondence X Anxious	0.01	0.01	−0.01, 0.01	0.427
Certainty	0.03	0.02	−0.01, 0.07	0.176
Avoidant	−0.22	0.04	−0.30, −0.15	0.001
Anxious	−0.07	0.02	−0.11, −0.02	0.004
Certainty X Avoidant	0.02	0.01	0.01, 0.04	0.017
Certainty X Anxious	0.02	0.01	0.01, 0.03	0.005
Mutual	−0.00	0.02	−0.03, 0.03	0.971
Avoidant	−0.23	0.04	−0.30, −0.15	0.001
Anxious	−0.07	0.02	−0.11, −0.02	0.004
Mutual X Avoidant	0.01	0.01	−0.01, 0.02	0.516
Mutual X Anxious	−0.01	0.01	−0.03, −0.01	0.010
Future	−0.02	0.02	−0.06, 0.01	0.256
Avoidant	−0.23	0.04	−0.30, −0.15	0.001
Anxious	−0.07	0.02	−0.11, −0.02	0.004
Future X Avoidant	−0.00	0.01	−0.02, 0.02	0.997
Future X Anxious	−0.01	0.01	−0.02, 00	0.078
Power	0.01	0.03	−0.04, −0.06	0.677
Avoidant	−0.23	0.04	−0.30, 0.15	0.001
Anxious	−0.07	0.02	−0.11, −0.02	0.004
Power X Avoidant	0.00	0.01	−0.02, 0.03	0.835
Power X Anxious	0.01	0.01	−0.01, 0.02	0.591

**Note:***b* = regression coefficient, SE = standard error; CI = confidence interval.

**Table 3:** Predicting reports of trust from levels of each interdependence feature, intake levels of attachment orientations, and their interactions.

	*b*	SE	95% CI	*p*
Correspondence	0.02	0.02	−0.02, 0.05	0.341
Avoidant	−0.16	0.03	−0.23, −0.09	0.001
Anxious	−0.10	0.02	−0.14, −0.05	0.001
Correspondence X Avoidant	0.02	0.01	0.01, 0.04	0.012
Correspondence X Anxious	0.00	0.01	−0.01, 0.01	0.868
Certainty	0.03	0.02	−0.01, 0.06	0.155
Avoidant	−0.16	0.03	−0.23, −0.09	0.001
Anxious	−0.09	0.02	−0.14, −0.05	0.001
Certainty X Avoidant	0.02	0.01	−0.01, 0.03	0.224
Certainty X Anxious	0.01	0.01	−0.00, 0.02	0.053
Mutual	0.01	0.02	−0.02, 0.04	0.425
Avoidant	−0.16	0.03	−0.23, −0.09	0.001
Anxious	−0.09	0.02	−0.14, −0.05	0.001
Mutual X Avoidant	−0.01	0.01	−0.02, 0.01	0.334
Mutual X Anxious	−0.01	0.01	−0.02, −0.00	0.033
Future	−0.01	0.02	−0.04, 0.02	0.674
Avoidant	−0.16	0.03	−0.23, −0.09	0.001
Anxious	−0.09	0.02	−0.14, −0.05	0.001
Future X Avoidant	−0.01	0.01	−0.03, 0.00	0.133
Future X Anxious	−0.01	0.01	−0.02, 0.00	0.101
Power	−0.00	0.02	−0.05, 0.04	0.967
Avoidant	−0.16	0.03	−0.23, −0.09	0.001
Anxious	−0.09	0.02	−0.14, −0.05	0.001
Power X Avoidant	0.01	0.01	−0.01, 0.04	0.296
Power X Anxious	−0.01	0.01	−0.02, 0.01	0.500

**Note:***b* = regression coefficient, SE = standard error; CI = confidence interval.

## Supplementary Materials

The following are available online at https://www.mdpi.com/1660-4601/17/20/7648/s1, Table S1: Correlations among interdependence dimensions in Experience Sampling, Table S2: Correlations among mean values of interdependence dimensions in the Experience Sampling, Table S3: Associations between interdependence dimension, attachment orientation, and their interactions for relationship satisfaction with all simultaneous dimensions in one model, Table S4: Associations between interdependence dimension, attachment orientation, and their interactions for trust with all simultaneous dimensions in one model.
