# Supplementary material for: Fostering Attachment Security: The Role of Interdependent Situations
Source: Int J Environ Res Public Health. 2020 Oct 20;17(20):7648. doi: 10.3390/ijerph17207648 (PMC7589934; doi:10.3390/ijerph17207648)
Supplement: Supplementary file 1 [file ijerph-17-07648-s001.pdf]

Supplemental Material

Table 1. Correlations among interdependence dimensions in Experience Sampling.

**Variables**	**(2)**	**(3)**	**(4)**	**(5)**
(1) Correspondance	0.37 **	-0.08 **	-0.04 **	0.05 **
(2) Certainty	—	-0.04 **	-0.06 **	0.05 **
(3) Mutual		—	0.43 **	-0.02
(4) Future			—	-0.01
(5) Power				—

Note. Values correspond to within-person centered variables; $^{*}p < .05$. $^{**}p < .01$. ***

Table 2. Correlations among mean values of interdependence dimensions in the Experience Sampling.

**Variables**	**(2)**	**(3)**	**(4)**	**(5)**
(1) Correspondance	0.73 **	-0.16 **	-0.21 **	0.10
(2) Certainty	—	-0.21 **	-0.22 **	0.10
(3) Mutual		—	0.85 **	-0.09
(4) Future			—	-0.08
(5) Power				—

Note. Values correspond to mean values of interdependence dimensions collected during the whole experience sampling; $^{*}p < .05$. ** $p < .01$.

Table S3. Associations between interdependence dimension, attachment orientation, and their interactions for relationship satisfaction with all simultaneous dimensions in one model

	b	***SE***	**95% CI**	***p***
Avoidant	-.23	.04	-.30, -.15	.001
Anxious	-.07	.02	-.11, -.02	.004
Correspondance	.04	.02	-.00, .08	.071
Certainty	.00	.02	-.04, .05	.700
Mutual	.02	.02	-.02, .06	.337
Future	-.03	.02	-.07, .01	.137
Power	-.00	.03	-.05, .05	.903
CorrespondanceXAvoidant	.01	.01	-.01, .03	.147
CorrespondanceXAnxiety	-.00	.01	-.01, .01	.841
CertaintyXAvoidant	.02	.01	-.00, .04	.095
CertaintyXAnxiety	.02	.01	.01, .03	.004
MutualXAvoidant	.00	.01	-.02, .02	.770
MutualXAnxious	-.01	.01	-.02, .00	.078
FutureXAvoidant	-.00	.01	-.02, .02	.997
FutureXAnxious	-.01	.01	-.02, 00	.078
PowerXAvoidant	.00	.01	-.02, .03	.474
PowerXAnxious	.00	.01	-.01, .02	.899

Table S4. Associations between interdependence dimension, attachment orientation, and their interactions for trust with all simultaneous dimensions in one model

	b	***SE***	**95% CI**	***p***
Avoidant	-.16	.03	-.23, -.09	.001
Anxious	-.09	.02	-.14, -.05	.001
Correspondance	.01	.02	-.03, .04	.706
Certainty	.02	.02	-.02, .06	.241
Mutual	.02	.02	-.01, .06	.148
Future	-.02	.02	-.05, .02	.303
Power	-.01	.02	-.06, .03	.611
CorrespondanceXAvoidant	.02	.01	.00, .04	.034
CorrespondanceXAnxiety	-.00	.01	-.01, .01	.660
CertaintyXAvoidant	.00	.01	-.02, .02	.910
CertaintyXAnxiety	.01	.01	.00, .03	.034
MutualXAvoidant	-.01	.01	-.02, .01	.455
MutualXAnxious	-.01	.01	-.02, .00	.173
FutureXAvoidant	-.01	.01	-.02, .01	.439
FutureXAnxious	-.01	.01	-.02, 01	.342
PowerXAvoidant	.02	.01	-.01, .04	.439
PowerXAnxious	-.01	.01	-.02, .01	.326

Note: The above model cannot be reliably estimate using the mean values of the interdependent dimensions due to collinearity problems between dimensions (see Table S2).
